# Genetic Transformation of *Triticum dicoccum* and *Triticum aestivum* with Genes of Jasmonate Biosynthesis Pathway Affects Growth and Productivity Characteristics

**DOI:** 10.3390/plants13192781

**Published:** 2024-10-04

**Authors:** Dmitry N. Miroshnichenko, Alexey V. Pigolev, Alexander S. Pushin, Valeria V. Alekseeva, Vlada I. Degtyaryova, Evgeny A. Degtyaryov, Irina V. Pronina, Andrej Frolov, Sergey V. Dolgov, Tatyana V. Savchenko

**Affiliations:** 1 Institute of Basic Biological Problems, Pushchino Scientific Center for Biological Research, Russian Academy of Sciences, 142290 Pushchino, Russia; alexey-pigolev@rambler.ru (A.V.P.); evkras99@yandex.ru (E.A.D.); savchenko_t@rambler.ru (T.V.S.); 2 Branch of Shemyakin–Ovchinnikov Institute of Bioorganic Chemistry, Russian Academy of Sciences, 142290 Pushchino, Russia; aspushin@gmail.com (A.S.P.); lera@bibch.ru (V.V.A.); vlada.buryak@gmail.com (V.I.D.); dolgov@bibch.ru (S.V.D.); 3 Department of Physiology, Human Ecology and Medical and Biological Sciences, State University of Education, 141014 Mytishi, Russia; zolly_sten@mail.ru; 4 Laboratory of Analytical Biochemistry and Biotechnology, Timiryazev Institute of Plant Physiology, Russian Academy of Sciences, 127276 Moscow, Russia; andrej.frolov@ipb-halle.de

**Keywords:** particle inflow gun, bread wheat, emmer wheat, jasmonates, allene oxide synthase, 12-oxophytodienoate reductase

## Abstract

The transformation protocol based on the dual selection approach (fluorescent protein and herbicide resistance) has been applied here to produce transgenic plants of two cereal species, emmer wheat and bread wheat, with the goal of activating the synthesis of the stress hormone jasmonates by overexpressing *ALLENE OXIDE SYNTHASE* from *Arabidopsis thaliana* (*AtAOS*) and bread wheat (*TaAOS*) and *OXOPHYTODIENOATE REDUCTASE 3* from *A. thaliana* (*AtOPR3*) under the strong constitutive promoter (*ZmUbi1*), either individually or both genes simultaneously. The delivery of the expression cassette encoding AOS was found to affect morphogenesis in both wheat species negatively. The effect of transgene expression on the accumulation of individual jasmonates in hexaploid and tetraploid wheat was observed. Among the introduced genes, overexpression of *TaAOS* was the most successful in increasing stress-inducible phytohormone levels in transgenic plants, resulting in higher accumulations of JA and JA-Ile in emmer wheat and 12-OPDA in bread wheat. In general, overexpression of *AOS*, alone or together with *AtOPR3*, negatively affected leaf lamina length and grain numbers per spike in both wheat species. Double (*AtAOS* + *AtOPR3*) transgenic wheat plants were characterized by significantly reduced plant height and seed numbers, especially in emmer wheat, where several primary plants failed to produce seeds.

## 1. Introduction

Plant hormones jasmonates are responsible for regulating stress responses, and the activation of jasmonate signaling may be an effective strategy to increase plant stress tolerance. The biosynthesis of plant hormone jasmonates is a well-studied process (reviewed in detail) [1]. Octadecatrienoic (18:3) acid and more rarely hexadecatrienoic (16:3) acid are known substrates for jasmonate biosynthesis. In chloroplasts, lipoxygenase, allene oxide synthase (AOS) (EC 4.2.1.92), and allene oxide cyclase form 12-oxo-phytodienoic acid (12-OPDA) from 18:3 or dinor-OPDA from 16:3 [2]. OPDA is then transported to peroxisomes, where the double bond in the cyclic part of the molecule is reduced by oxophytodienoate reductase (OPR) (EC 1.3.1.42), and after side chain shortening through the β-oxidation, jasmonic acid (JA) is formed. Further modifications of JA occur in the cytoplasm, including the formation of the bioactive ligand jasmonate-isoleucine (JA-Ile).

The first components of the jasmonate signaling system emerged in the last common ancestor of land plants, and the presence of genes for all of the jasmonate biosynthesis enzymes and the components of the signal transduction pathway has been confirmed in all studied monocotyledonous and dicotyledonous plants [3,4,5]. At the same time, there is an increasing body of evidence indicating that the jasmonate systems of monocotyledonous and dicotyledonous plants exhibit distinct differences [6,7,8]. One notable example is the uncovered distinct functions of the two receptors for JA-Ile in rice, namely, canonical and non-canonical, which suggest a sub-functionalization of the jasmonate receptors in the monocot phylum, whereas a single JA-Ile receptor is in the dicotyledonous Arabidopsis [9,10]. Another example of substantial differences from dicotyledons was demonstrated in maize by showing that 12-OPDA and a-ketol of octadecadienoic acid, but not JA or JA-Ile, are signals required for *Trichoderma virens*-induced systemic resistance in this plant [11]. In comparison to Arabidopsis, wheat exhibited a markedly lower tissue jasmonate content and a very limited inducibility of hormone production by mechanical damage of the tissue [12], even though the content of octadecatrienoic acid in wheat leaves, a substrate of jasmonate biosynthesis, is known to be very high, even higher than in Arabidopsis [13]. In general, there is a clear need for further studies of the jasmonate system in different representatives of the monocotyledons.

The AOS that catalyzes the first committed step in the biosynthesis of jasmonates has attracted significant research interest. The Arabidopsis genome contains a single *AOS* gene, while the rice genome contains four, although only two of them have been demonstrated to be involved in JA biosynthesis, tolerance to insects and bacterial blight, and the protection of seedlings from arsenic [14,15,16,17]. There are at least two *AOS* genes in barley [18]. Multiple *AOS* genes were identified in the genomes of wild and tropical species of sugarcane—eight and 36 *AOS* genes, respectively. Among the encoded enzymes, there are enzymes specific to 13-hydroperoxides and 9-hydroperoxides as well as the mixed 9-/13-AOS type [19]. In bread wheat, 12 putative *AOS* genes, represented by four triads of homoeologous genes, were identified [20]. In *T. aestivum*, *AOS* is highly expressed in flag leaves, and its expression is induced in response to salts and powdery mildew fungi [21]. The overexpression of this gene in tobacco enhances the plant’s tolerance to excessive zinc concentrations. In the roots of wheat, barley, sorghum, and rice, AOS also catalyzes the formation of minor products, including cyclopentenone 12-oxo-10,15-phytodienoic acid, and other oxylipins collectively named “graminoxins”, whose biological functions remain unknown [22].

The *OPR* genes have been the subject of comparatively little study in monocotyledons, particularly with regard to their biological functions. In the Arabidopsis genome, there are three *OPR* genes (*OPR1*, *OPR2*, and *OPR3*). OPR3 is the main enzyme of JA biosynthesis [23], although the contributions of other genes to jasmonate biosynthesis have been demonstrated [24]. Among the ten *OPRs* in rice, *OsOPR7* shares the highest sequence identity with *AtOPR3* and plays a dominant role in JA production [25]. Two *OPR* genes, *ZmOPR7* and *ZmOPR8*, were shown to be involved in JA biosynthesis in maize [26]. Genome-wide bioinformatic analysis revealed forty-eight putative *OPR* genes in the wheat species *T. aestivum*, which were classified into five subfamilies [27]. However, the biological functions of these genes in wheat remain uncharacterized to date.

The hexaploid bread wheat (*T. aestivum* L., 2n = 6x = 42) and the tetrapoloid emmer wheat (*Triticum dicoccum*; Schrank., 2n = 4x = 28 [syn *Triticum turgidum* L. *subsp. dicoccum* (*Schrank ex Schubl*.) *Thell*]) have been cultivated for centuries by human population as food, feed, and industrial crops. In recent years, jasmonate biosynthesis in wheat has emerged as a subject of considerable interest within the scientific community, with implications for both fundamental and practical insights [12,13,21,22,27]. In our recent study, we generated transgenic emmer wheat plants that overexpress either *ALLENE OXIDE SYNTHASE* (*AtAOS*) or *OXOPHYTODIENOATE REDUCTASE 3* (*AtOPR3*) from *Arabidopsis thaliana* for the first time [12,28]. Previously, we also demonstrated the successful overexpression of the *AtOPR3* gene in bread wheat [29]. These studies showed that the overexpression of jasmonate biosynthesis pathway genes leads to the alteration of plant developmental traits and stress tolerance and represents a reliable tool for gaining a better understanding of the jasmonate system in wheat [12,28,29,30]. The present study aimed to generate a new panel of transgenic wheat plants overexpressing the *AOS* and *OPR* genes. For the first time, transgenic plants of tetraploid and hexaploid wheat varieties that simultaneously overexpress the *OPR* gene, encoding a peroxisome-localized enzyme, and the *AOS* gene, encoding a protein localized in chloroplasts, have been produced. We also compared the genetic transformation of wheat varieties with the heterologous *AOS* gene from Arabidopsis and the endogenous *AOS* gene. The effects of the delivery and expression of cassettes encoding jasmonate biosynthetic genes are analyzed and discussed in various aspects, including the efficiency of the genetic transformation and changes in hormone levels, plant development, and productivity.

## 2. Results

### 2.1. Generation of Emmer Wheat and Bread Wheat Plants Co-Expressing AtAOS and AtOPR3 Genes

The biolistic-mediated gene transfer approach allows the simultaneous introduction of different expressing cassettes into the plant genome by the bombardment of recipient cells with a mix of various plasmids. To overexpress the *AtAOS* and *AtOPR3* genes in the same wheat plant, we co-delivered the pBAR-GFP.UbiAOS [28] and pBAR-GFP.UbiOPR3 [29] plasmids into the 301 morphogenic calli of the emmer wheat Runo. As a result of selection, 20 GFP positive plantlets were recovered from ten explants (one to six plantlets per individual explant) and transferred to soil. PCR analysis confirmed that all of the greenhouse-grown plants were transgenic, as they carried at least the sequence of the GFP reporter gene in DNA samples extracted from their leaves (Figure S1A). Most of the events (plants grown from the six initial explants) displayed successful co-integration of both the *AtAOS* and *AtOPR3* genes from different plasmids (Figure S1A). The rest of the transgenic plants (recovered from four initial explants) showed only amplification of the *AtOPR3* specific fragment (Figure S1A). End-point RT-PCR of leaf RNA extracts (Figure S1B) of the greenhouse-grown plants revealed the accumulation of both *AtOPR3* and *AtAOS* transcripts in three primary transgenic events, labeled RAB1, RAB2, and RAB5, including all of the ‘twin’ transgenic plants recovered from initial explants. One event, RAB9, showed only an accumulation of *AtAOS* transcripts. The two other events, RAB6 and RAB7, demonstrated overexpression of the *AtOPR3* gene only (Figure S1B).

**Figure 1:**
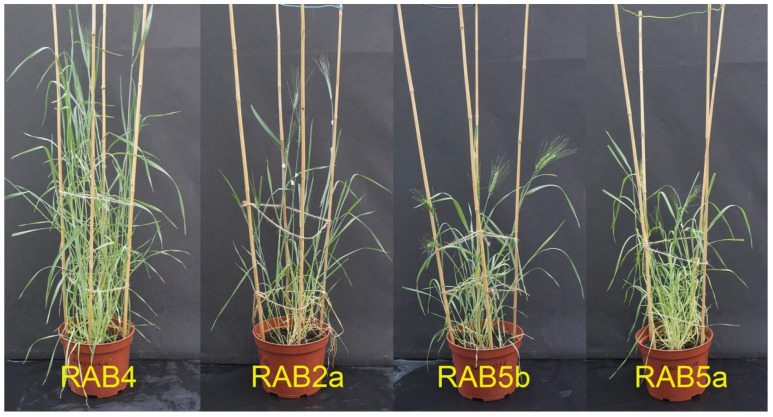
Emmer wheat plants transformed with *AtAOS* and *AtOPR3* genes around the flowering stage as grown in the greenhouse; note developmental differences between the primary T0 plants, RAB4, which is silenced for expression of introduced genes and the RAB2a, RAB5a, and RAB5b plants with a high level of constitutive expression of both the *AtAOS* and *AtOPR3* genes.

During the cultivation of double transgenic plants of emmer wheat, we discovered the significant retardation of growth in RAB2 and RAB5 primary T0 plants (Figure 1). Such behavior was similar in all of the ‘twin’ T0 plants (3 plants of RAB2 and 5 plants of RAB5) recovered from the initial explants at different times of selection. The other T0 plants, including the double gene expressing RAB1, single gene expressing RAB6, RAB7, RAB9, and RAB4, which did not express the introduced genes, did not show such significant deviations. Our previous observations showed that the complexity of recovering transgenic plantlets in tissue culture and the seasonal effects of ex vitro adaptation may influence the growth parameters of transgenic plants. To correctly analyze the effect of the introduced genes, T1 seeds had to be produced so that we could select homozygous T2-T4 transgenic progenies with stably inherited foreign gene expression. In this instance, we encountered an issue related to seed set in RAB2 and RAB5 primary plants. None of the RAB5 primary plants produced seeds. A single T1 seed was produced by three T0 plants of the RAB2 events; however, this seed failed to germinate. At the same time, the double primary transgenic plant RAB4, which is silenced for the expression of *AtOPR3* and *AtAOS* genes, grown in a greenhouse in parallel with RAB2 and RAB5 plants, yielded the normal seed set (>300 seeds per plant).

**Figure 2:**
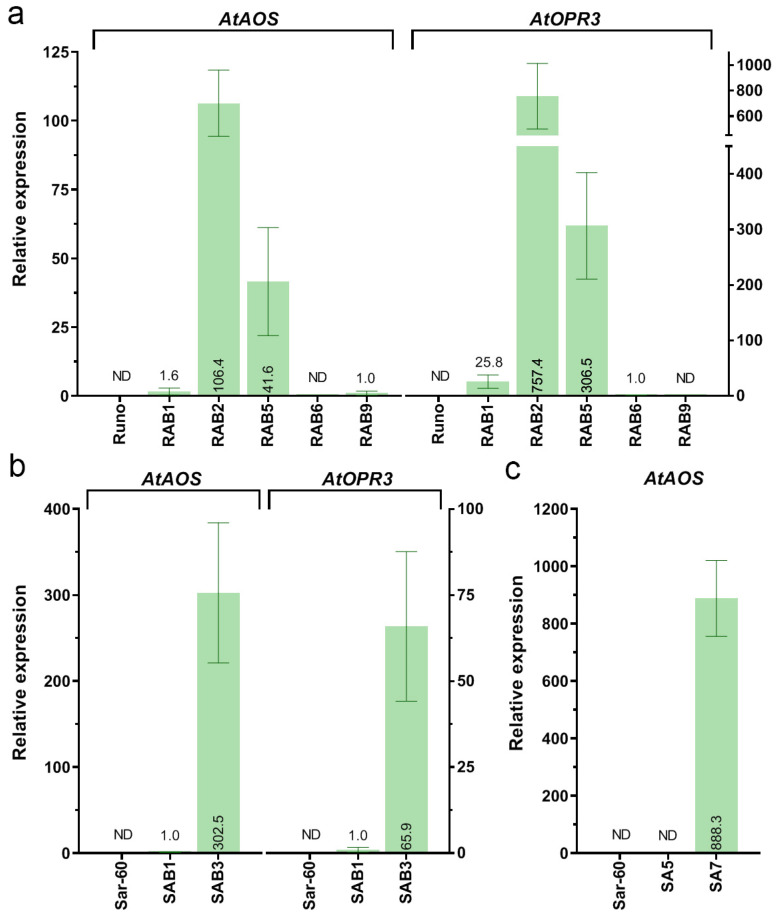
Relative expression levels of *AtAOS* and *AtOPR3* in leaves of transgenic emmer (**a**) and bread (**b**) wheat lines; T4 homozygous plants, with the exceptions of RAB2 and RAR5, where leaf extracts of T0 plants are analyzed; data are means of at least three biological replicates ± SE; (**a**,**b**) expression levels in plants of ‘double’ transgenic lines carrying *AtAOS* and *AtOPR3* genes; (**c**) expression levels of *AtAOS* gene in transgenic lines of bread wheat Sar-60, for normalization, the relative expression level detected in SAB1 plants (panel (**b**)) is used.

To confirm that the transferred genes were expressed in the offspring, the selected homozygous T4 sub-lines of primary events RAB1, RAB6, and RAB9 were analyzed by qRT-PCR. The analysis also included samples of total RNA extracted from the leaves of the primary RAB2 and RAB5 events. As the parent emmer wheat, Runo, does not express *AtAOS* or *AtOPR3*, the lowest accumulation of transcripts detected by qRT-PCR in transgenic samples of RAB9 and RAB6 was taken as “1” to quantify the expression levels of *AtAOS* and *AtOPR3*, correspondingly. These expression data (Figure 2a) showed that both transgenes were markedly overexpressed in the seedless RAB2 and RAB5 plants. We found 25-fold and 60-fold more transcripts of *AtAOS* in the leaves of RAB5 and RAB2 plants than in the RAB1 plants that also expressed both genes. Similarly, the expression of *AtOPR3* in RAB5 and RAB2, correspondingly, was 10-fold and 25-fold higher than in the RNA extracts from RAB1 plants. As expected, the progenies of RAB9 showed only expression of *AtAOS*, while in the RNA extracts of homozygous RAB6 plants, only *AtOPR3* transcripts were detected (Figure 2a).

In a parallel experiment, 358 embryo-derived calli of the bread wheat Sar-60 were bombarded with a mix of pBAR-GFP.UbiAOS and pBAR-GFP.UbiOPR3 plasmids. Four putative transgenic plants were recovered from three initial explants under rounds of herbicide+GFP selection. The introduction of both genes, *AtAOS* and *AtOPR3*, was found in three plants using PCR analysis (Figure S2A); two of them were regenerated from the same explant and later were regarded as the ‘twin’ plants of the same transformation event. The end-point RT-PCR analysis on the total RNA of T0 plants at the heading stage confirmed the overexpression of the foreign genes, including all of the double transformants (Figure S2B).

The double positive events SAB1 and SAB3 were morphologically normal, fertile, and successfully produced T1 seeds. Analysis of 49 SAB1 and 74 SAB3 individual T1 plants allowed us to select for each primary event homozygous sub-lines with stable inheritance of the *GFP* marker transgene in their T2-T4 progenies. Using the cDNA as the template for qRT-PCR, the transcript accumulation of both *AtOPR3* and *AtAOS* was also successfully detected in foliage RNA extracts of T4 homozygous lines of SAB1 and SAB3. As expected, no expression of foreign genes was found in non-transgenic plants of the bread wheat Sar-60 (Figure 2b).

#### 2.1.1. Integration and Overexpression of the *AtAOS* Gene in Bread Wheat

Previously, we successfully introduced the *ALLENE OXIDE SYNTHASE* gene from *Arabidopsis* (*AtAOS*) into the genome of emmer wheat [28]. The same transformation methodology was used here to generate transgenic plants of the bread wheat Sar-60 using the pBAR-GFP.UbiAOS vector. Of the 717 bombarded explants, nine putative transgenic events were recovered. Genomic PCR analysis of mature greenhouse-grown plants revealed that one of the events was an escape (Figure S3A). Among the eight transgenic events, the SA3 plant showed the silencing of *GFP* expression, which was further confirmed by the absence of green fluorescence in the pollen and was consistent with the results of the RT-PCR analysis (Figure S3B). According to RT-PCR analysis, half of the primary transgenic events failed to accumulate the transcripts of the *AtAOS* gene (Figure S3B). The four *AtAOS* positive T0 events were further analyzed for the inheritance of transgenes in their T1-T2 offspring. Only two of them (SA5 and SA7) showed a Mendelian ratio (3:1) of transgene inheritance that allowed the identification of the stable homozygous sub-lines with a single insertion of the transgene. Expression analysis, evaluated by qRT-PCR (Figure 2c), indicated a significant abundance of the *AtAOS* transcript in the leaves of the SA7 line (T4 plants). The introduced *AtAOS* gene was not transcribed in the T4 homozygous plants of the SA5 line, and, as expected, in the non-transgenic parent plants (Figure 2c).

#### 2.1.2. Generation of Transgenic Emmer Wheat and Bread Wheat Plants Overexpressing *ALLENE OXIDE SYNTHASE* (*TaAOS*) from Bread Wheat

The other transformation experiment aimed to overexpress the endogenous *ALLENE OXIDE SYNTHASE* (*TaAOS*) gene constitutively in both emmer wheat and bread wheat. A “short” variant of the *AOS* gene from the wheat genome “A” (TraesCS4A02G061800.1), also known as *TaAOS2* [15,31], was chosen for overexpression because according to our previous study, the expression of this variant is enhanced by plant injury. Like the Arabidopsis *AOS* gene, the wheat *AOS* gene lacks introns. Therefore, the *TaAOS* gene sequence was obtained by PCR from the genomic DNA of Sar-60. To facilitate gene expression in plants, the Kozak sequence for monocotyledons was incorporated upstream of the translation initiation codon. In combination with a strong constitutive promoter from maize (*ZmUbi1*), this should allow robust expression of the transgene.

**Figure 3:**
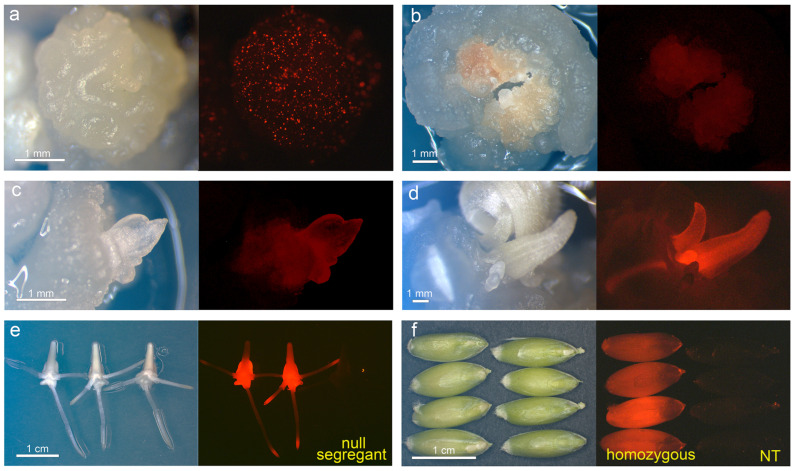
Production of transgenic wheat plants constitutively overexpressing *TaAOS* gene. (**a**) Transient *RFP* gene expression; morphogenic explant 24 h after the delivery of pANIC-*TaAOS* plasmid to Runo cells; (**b**) aging and necrosis of Runo wheat tissue with *RFP* expression; 45 days of in vitro culture; (**c**) early stage of transgenic somatic embryo formation of emmer wheat Runo, 60 days after bombardment with decreased concentration of herbicide; (**d**) formation of the RFP-positive single embryo-like structure of Sar-60 surrounded by leafy structures with RFP fluorescence on the medium with decreased herbicide concentration, 80 days after bombardment; (**e**) segregation of introduced expression cassette in T1 embryos germinated in vitro; 5 days of culture; transgenic line SD3 (**f**) RFP fluorescence in T2 kernels of homozygous sub-line RD1 in comparison with non-transgenic kernels of emmer wheat Runo. Bright field images are shown on the left side and fluorescent images are shown on the right side.

A dual selection approach (herbicide resistance and Red Fluorescent Protein (RFP) expression in selected cells) was used to generate transgenic plantlets. Initially, explants subjected to the bombardment with pANIC-*TaAOS* showed the formation of multicellular loci exhibiting distinctive *RFP* expression (Figure 3a). However, starting from the second sub-culture, the massive death of morphogenic structures was observed, caused by rapid aging and necrosis of the tissues that included the *RFP* expressing structures (Figure 3b). As a result, the first transformation experiments failed to produce transgenic plants. In the next experiments, to weaken selective pressure and thereby reduce the stress load on the plants, the concentration of phosphinothricin (PPT) in the medium was reduced from 5 to 2 mg/L. Following the decrease in herbicide concentration, a few plantlets with detectable levels of red fluorescence were recovered in both tetraploid and hexaploid wheat (Figure 3c,d). Despite the increased number of bombarded explants (547 calli for Runo, 1013 calli for Sar-60), only three independent events were recovered in each wheat cultivar. The integration of *RFP* and *TaAOS* sequences was found in all six transgenic T0 plants using PCR (Figure S4).

All of the primary transgenic plants grew successfully to maturity with no visible abnormalities during development. To speed up the segregation analysis, the immature T1 embryos from the ears of each primary event were isolated and germinated in vitro while monitoring RFP fluorescence. Such an approach allowed us to identify the T1 seedlings that inherited functional loci of the introduced expression cassette (Figure 3e). Unfortunately, the T1 embryos of primary event RD2 showed no fluorescent activity and thus were omitted from the further research. The rest of the five T0 transgenic events showed variable patterns of transgene inheritance (with segregation from 1:1 to 3:1). After the T1 seedlings positive for red fluorescence were grown to maturity, the homozygous sub-lines were detected (Figure 3f) in two independent lines of bread wheat (SD2 and SD3) and two lines of emmer wheat (RD1 and RD4).

**Figure 4:**
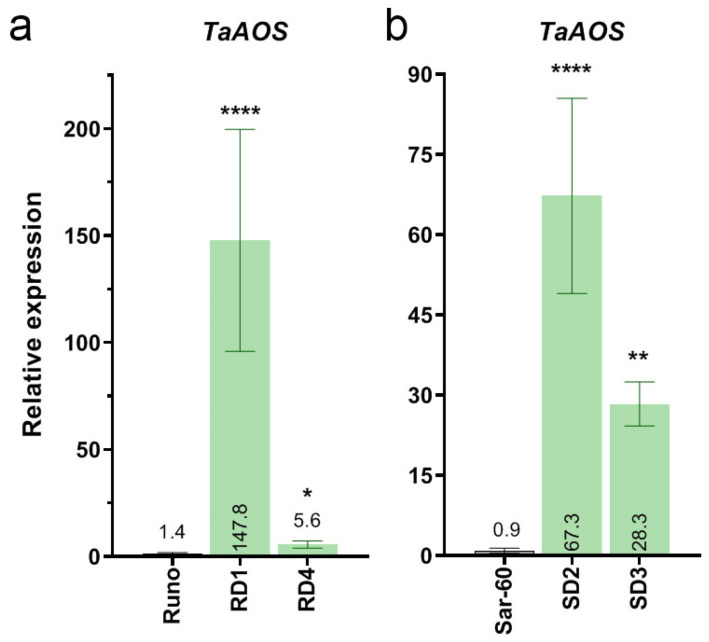
Expression levels of *TaAOS* gene in leaves of transgenic wheat lines; (**a**) emmer wheat (cv. Runo) transgenic lines; (**b**) bread wheat (Sar-60) transgenic lines; data are means of at least five biological replicates ± SE; stars above the graphs indicate statistically significant differences with non-transgenic wheat (* *p* ≤ 0.05, ** *p* ≤ 0.01, **** *p* ≤ 0.001).

The level of *TaAOS* expression in transgenic lines was checked using a real-time PCR assay using leaf RNA extracts of the T4 homozygous plants (Figure 4). Compared to the non-transgenic plants, the transcripts of the gene encoding *TaAOS* were increased in all of the analyzed homozygous sub-lines positive for *RFP* expression. In particular, the mRNA level of *TaAOS* in transgenic plants of RD1 was strongly increased by 100-fold. In the leaves of the RD4, SD3, and SD2 lines, the accumulation of *TaAOS* transcripts was higher by 4, 35, and 75-fold, respectively, compared to the corresponding control plants.

### 2.2. Characterization of the Transgenic Wheat Plants

#### 2.2.1. Phytohormone Analysis

To evaluate the effect of the transferred genes on the levels of jasmonates in the transgenic plants, we measured the content of 12-OPDA, JA, and JA-Ile conjugate in intact and mechanically wounded plant leaves. Similarly to our previous works on the study of jasmonates in wheat plants [12,27], we observed a rather large variation in hormone levels between individual plants, which resulted in a lack of statistically significant differences from non-transgenic plants for the majority of the transgenic lines, although the trend for an increase in the content of all measured jasmonates in intact or/and wounded leaves was observed in most transgenic lines (Table 1; Figure S5). An increase in jasmonate content after leaf wounding is also evident for the non-transgenic and transgenic lines. Statistically significant alterations in levels of measured jasmonates were detected only in the lines overexpressing *AOS* from wheat, including the emmer wheat lines RD1 and RD4 and the bread wheat lines SD2 and SD3 (Table 1). Interestingly enough, in hexaploid wheat, overexpression of *TaAOS* led to an increase in only the 12-OPDA levels in both intact and wounded leaves, while JA and JA-Ile levels remained unaltered. In tetraploid emmer wheat, by contrast, an increase in wounding-induced levels of JA and JA-Ile was observed while 12-OPDA levels remained unchanged. Also of interest is the decreased basal level of JA and JA-Ile in both hexaploid and tetraploid wheat overexpressing *TaAOS*, with these changes being statistically significant in the RD1 and SD3 lines.

Although no statistically significant differences were found in the content of the metabolites analyzed, the transgenic lines overexpressing *AtAOS* (SA7) and *AtAOS* simultaneously with *AtOPR3* (SAB1), on average show very high mean 12-OPDA values in wounded leaves, comparable to the values observed in SD2 and SD3 (Figure S6). On average, the 12-OPDA levels in damaged leaves of SA7 and SAB1 exceed these values in non-transgenic Sar-60 by approximately 2.4- and 5.3-fold, respectively. The wounding-induced levels of JA-Ile on average also increased 1.5 times in the SAB1 line.

As noted earlier, Runo plants RAB2 and RAB5, overexpressing two genes, *AtAOS* and *AtOPR3*, did not produce progeny. We had at our disposal only frozen leaves of the T0 plants at a late stage of development, which we used to analyze transgene expression and the basal level of jasmonic acid. The different physiological states of the plants from which these tissues were collected do not allow the comparison of the results obtained with other data. A comparative analysis of the JA content of intact leaves of the transgenic plants and non-transgenic controls revealed a notably decreased JA level in RAB2, which is analogous to the reduction in jasmonates observed in RD1 and SD3 (Figure S6). In RAB1 and RAB5, the basal JA level is the same as in Runo plants of the same age.

#### 2.2.2. Analysis of the Growth of Transgenic Plants

**Figure 5:**
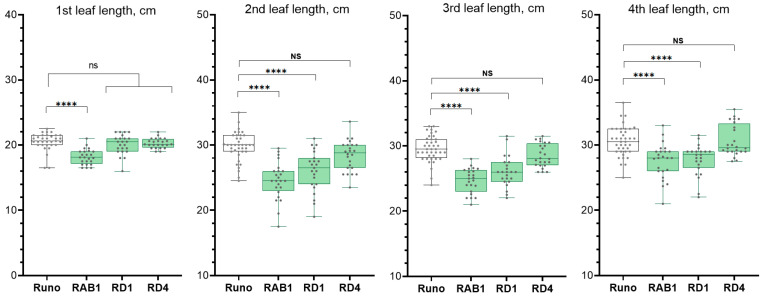
Analysis of leaf length of non-transgenic emmer wheat (cv. Runo) and transgenic plants with overexpression of *AtAOS* and *AtOPR3* (RAB1) or *TaAOS* (RD1 and RD4). Values represent the lengths of 1st, 2nd, 3rd, and 4th leaves measured in 22–25 plants (transgenic lines) or 38 plants (non-transgenic (Runo)) (average ± sd). Stars indicate statistically significant differences calculated according Dunnett’s multiple comparison test: (“****”, *p* < 0.001), (NS, non-significant).

**Figure 6:**
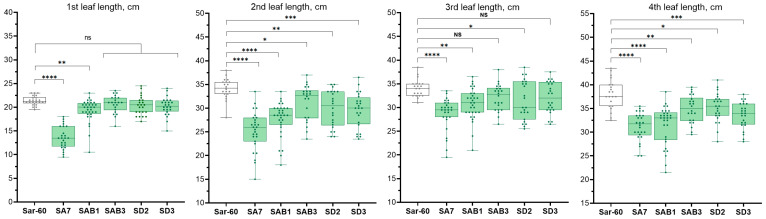
Analysis of leaf length of non-transgenic bread emmer wheat Sar-60 and transgenic lines with overexpression of *AtAOS* (SA7), *TaAOS* (SD2, SD3), or *AtAOS* and *AtOPR3* simultaneously (SAB1, SAB3). Values represent the lengths of 1st, 2nd, 3rd, and 4th leaves measured in 22–25 plants (average ± sd). Stars indicate statistically significant differences calculated according to Dunnett’s multiple comparisons test (“*”, *p* < 0.05), (“**”, *p* < 0.01), (“***”, *p* < 0.005), (“****”, *p* < 0.001), (NS, non-significant).

To examine the influence of the overexpression of *AtAOS*, *AtOPR3*, and *TaAOS* genes on their growth characteristics, T4 transgenic wheat plants were grown in a climate-controlled greenhouse and the length of four first fully developed leaves and the final plant height were measured. The measurement revealed that the overexpression of *ALLENE OXIDE SYNTHASE* generally led to the shortening of leaf length in both bread and emmer wheat. The most significant reduction was observed in the RA7 line (overexpressing *AtAOS*), and the two ‘double’ lines SAB1 and RAB1 (overexpressing *AtAOS* together with *AtOPR3*). The lengths of the 1st, 2nd, 3rd, and 4th leaves of these lines were significantly shorter by 10.2–36.5% (at *p* < 0.001) compared to the non-transgenic parent plants (Figure 5 and Figure 6).

In other *AOS* overexpressing lines, including the bread wheat (SAB3, SD2, SD3) and emmer wheat (RD1) transgenic lines, the shortening was less pronounced. In the early stages, plant growth was not different; the length of the 1st leaf was similar between transgenic and non-transgenic plants (Figure 5 and Figure 6). However, as they continued to grow, the plants of SAB3, SD3, SD2, and RD1 produced shorter 2nd, 3rd, and 4th leaves compared to the control plants (at *p* < 0.05 to *p* < 0.001). The only exception was the transgenic line RD4. The measurements from the first to the fourth leaf of transgenic plants of the RD4 line showed minor non-significant changes in the leaf length in comparison with the non-transgenic emmer wheat Runo (Figure 5).

**Figure 7:**
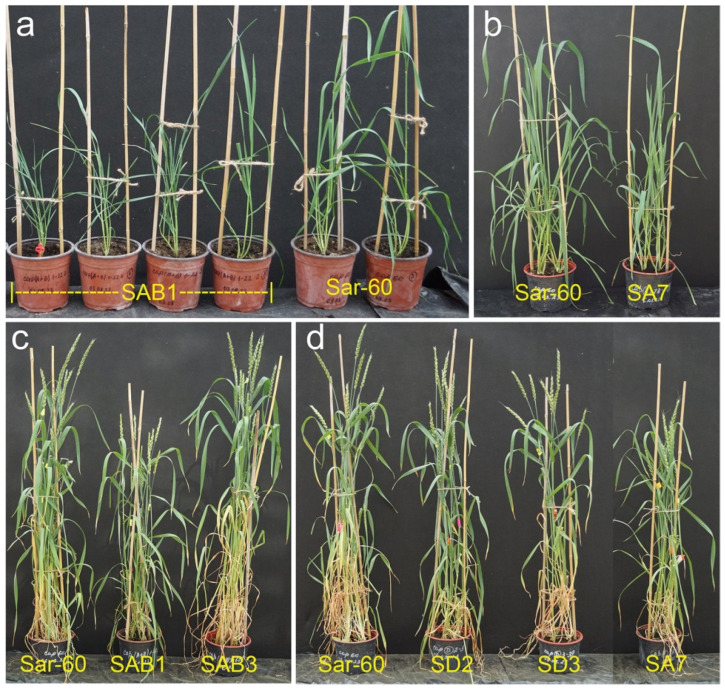
The morphology of transgenic bread wheat lines of plants transformed with *AtAOS* (SA7), *TaAOS* (SD2, SD3) and with both *AtAOS* and *AtOPR3* (SAB1, SAB3) genes. (**a**,**b**) plants are in boot developmental stage; (**c**,**d**) plants are in early ripening developmental stage.

**Figure 8:**
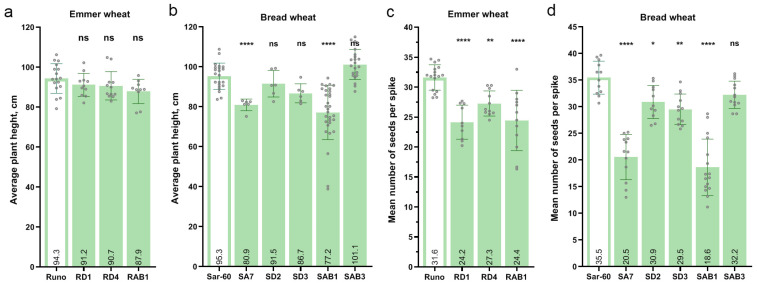
Average plant height and the productivity of transgenic wheat plants transformed with *AtAOS* (SA7), *TaAOS* (RD1, RD4, SD2, SD3), and *AtAOS* and *AtOPR3* simultaneously (RAB1, SAB1, SAB3). (**a**,**b**), average plant height; (**c**,**d**), mean number of seeds per spike; stars indicate statistically significant differences with corresponding non-transgenic wheat cultivar calculated according to Dunnett’s multiple comparisons test (“*”, *p* < 0.05), (“**”, *p* < 0.01), (“****”, *p* < 0.001), (ns, not significant).

The two transgenic lines of bread wheat, SA7 and SAB1, showed a significant delay in plant development at the later growth stages. A significant portion of the transgenic plants of SAB1 displayed a variable degree of delayed growth and reduced biomass (Figure 7a). The SA7 transgenic plants were also characterized by a similar reduction in growth (Figure 7b). This resulted in a significant reduction of the final plant height of SA7 and SAB1 plants (Figure 7c,d). On average, the plant height of the SA7 line was decreased by 14% (80.52 cm) relative to non-transgenic plants (95.2 cm) (Figure 8b). The plants of SAB1 were on average 18.4 cm shorter than control plants of Sar-60 (19% decrease in plant height). According to the ANOVA analysis, no significant differences in plant height were found between the non-transgenic parent cultivars and the other transgenic lines (Figure 8a,b).

As a result of the growth delay, both transgenic lines SA7 and SAB1 of the bread wheat produced a significantly lower number of seeds (Figure 8c,d). The average seed number collected per one spike of SA7 and SAB1 was decreased by 1.9-fold (18.6 seeds/spike) and 1.7-fold (20.5 seeds/spike), respectively, compared to the non-transgenic plants of Sar-60 (35.5 seeds/spike). In general, despite the absence of evident growth delays in other transgenic lines, the overexpression of the *AtAOS* gene, separately or together with the *AtOPR3* gene, led to a decrease in the number of seeds in most plants of both species (Figure 8c,d). All of the transgenic lines overexpressing the *TaAOS* gene had significantly lower seed numbers. Compared to 34.7 seeds per spike in non-transgenic Runo, the mean seed number in lines RD1 and RD4 was 27.7 (24% reduction, *p* < 0.005) and 28.6 (14% reduction, *p* < 0.05), respectively (Figure 8c). In the bread wheat transgenic lines SD2 and SD3, the average seed number differed from the non-transgenic Sar-60 by 12.9% (30.9 vs. 35.5, *p* < 0.05) and 16.8% (29.5 vs. 35.5, *p* < 0.01), respectively (Figure 8d). The transgenic emmer wheat line RAB1, simultaneously overexpressing both the *AtAOS* and *AtOPR3* genes, also showed a significant reduction in seed number (23%, *p* < 0.005). Despite a tendency for a reduction in the average number of harvested seeds in the bread wheat line SAB3, also overexpressing two genes, the difference was not significant according to the ANOVA test (*p* = 1.119) (Figure 8d).

## 3. Discussion

The first transgenic wheat plants were produced more than 40 years ago using high-velocity bombardment of targets with DNA-coated microprojectiles [32]. Since then, appreciable progress has been achieved in wheat transformation, mediated by both gene guns and *Agrobacterium* [33,34]. Despite the advancements that have already been made, the production of stable transgenic wheat lines carrying various genes of interest remains a challenging task. The underlying reasons are the complex genome and polyploid nature of modern wheat species. Such features largely predetermine the strict genotype dependence, low and unstable transformation, chromosome-position effects, multiple copy insertions, and the tendency for gene silencing in both primary events and the next generations. In line with the biotech trends of the last decades, we have developed a dual selection approach for the generation of primary transgenic plants of wheat species by manipulating various biological and physical factors during a biolistic-mediated transformation [35]. Screening for transgenic tissues using visual GFP observations coupled with herbicide-resistance selection was efficient for genetic transformation and genome editing of emmer wheat, bread wheat, and triticale [12,28,36,37]. Two wheat cultivars used in the present study showed no difficulties with biolistic-mediated transformation using a cassette carrying the *GFP* and *BAR* genes. The average genetic transformation rate of cv. Sar-60 (the hexaploid bread wheat) and cv. Runo (the tetraploid emmer wheat) was 2.3% and 13.0%, respectively (Table 2). When the pBAR-GFP expression cassette carrying the sequence of the *AtOPR3* gene was transferred to morphogenic cells of Sar-60 and Runo, the transformation efficacy was comparable with the empty vector, as the introduction rate of the gene of interest reached 12.7% in emmer wheat and 4.0% in bread wheat.

Unexpectedly, when we tried to overexpress another gene connected to JA biosynthesis, *AOS*, significant reductions in the transformation efficiency were found in both of the polyploid wheat cultivars. In the present study, the production rate of transgenic plants harboring the *AtAOS* gene decreased to 1% in bread wheat cv. Sar-60. Previously, a similar trend was also observed in emmer wheat cv. Runo, as the transformation rate with a vector containing the *AtAOS* sequence dropped to 1.7% [12]. Moreover, 40–47% of primary wheat events with confirmed insertions of *AtAOS* gene were silenced for expression. This contrasts with the transformation with the *AtOPR3* gene, where a significant part of the primary wheat events (66–87%) displayed the accumulation of transcript of the introduced transgene [29]. The difficulty was also evident in the case of co-delivering the *AtOPR3* gene together with the *AtAOS*. Co-bombardment by the mix of equal volumes of pBAR-GFP.UbiAOS and pBAR-GFP.UbiOPR3 constructs resulted in a transformation efficiency of 2.0% in emmer wheat and 0.8% in bread wheat (Table 2). Our attempts to achieve constitutive expression of the endogenous *TaAOS* gene in wheat also encountered difficulties, despite increasing the numbers of bombarded embryos. The constitutive expression of the *TaAOS* gene in transgenic cells appeared to have hindered the growth of the transformed calluses (Figure 3b), reducing transformation efficiency. As a result, the efficiency of the generation of transgenic plants was the lowest among all experiments. In comparison with the empty vector psGFP-BAR, the transformation rate decreased 9-fold in bread wheat (0.3%) and 25-fold in emmer wheat (0.5%) when a callus was transformed with the *TaAOS* gene. Only a few plants were produced after the weakening of selective pressure in a regeneration medium (Table 1).

The difficulties encountered in the production of transgenic wheat plants due to transferring the gene of interest are rarely reported. The challenges associated with the regeneration or even the failure to obtain transgenic plants have been mainly observed in studies aimed at a knock-down of the expression of specific endogenous genes in wheat plants. For example, a strong negative effect on the development of transgenic somatic embryos and plants was found after a transferring of RNAi cassettes into cells of highly transformable cultivars of wheat due to a post-transcriptional silencing of the glutathione biosynthesis (*GSH1* and *GSH*) genes [38], the grain hardness gene puroindoline (*Pinb*) [39], and glucan synthase-like (*TaGSL*) genes [40]. There is a report indicating that the overexpression of the *DELAY of GERMINATION* (*DOG*) genes resulted in a genetic transformation of low efficiency in bread wheat cv. Fielder, which is known for its high transformation abilities [41]. As in our study, the overexpression of the wheat *DOG* sequence was reported to be much more problematic than the overexpression of the heterologous homolog gene from another plant species (0.8% vs. 6.2%), while in both constructs the same *Ubi1* promoter was used to drive expression [41].

Since we used the *Ubi1* promoter to overexpress all the transferred genes (*AtAOS*, *AtOPR3*, and *TaAOS*) in the present study, it could be speculated that the reduction in transformation efficiency during the wheat’s transformation with *AOS* genes is not associated with the design of the expression cassettes. In our opinion, a replacement of the green fluorescent protein (GFP) gene in pBAR-GFP.UbiAOS with the Red Fluorescent Protein (RFP) gene in the pANIC-AOS vector could hardly have had a negative effect on the transformation efficiency. In several studies, the detection of transgenic tissue by RFP enabled the generation of transgenic wheat plants without any significant difficulties [42,43,44,45]. Our study shows that the use of RFP was equally helpful for the generation of transgenic plants in both emmer wheat and bread wheat (Figure 3). Based on the results obtained, we believe that the combination of the visual selection of *RFP* and *GFP* genes could be further useful for co-transformation experiments in wheat, where different genes of interest are placed on separate plasmids carrying different visual selection genes.

Our results indicate that an abundance of allene oxide synthase, the key JA synthesis enzyme, negatively affected the transformation rate in wheat. This becomes even more obvious when comparing the *OPR* and *AOS* genes from Arabidopsis. The design of the pBAR-GFP.UbiOPR3 and pBAR-GFP.UbiAOS vectors is completely identical, but only the presence of the *AOS* sequence in the vector has a negative effect on plant transformation and regeneration. Moreover, the up-regulation of the wheat *AOS* gene has an even stronger effect. Due to the transient/stable overexpression of the *TaAOS* sequence, mostly unhealthy calluses were observed. These were incapable of somatic embryogenesis, the main morphogenic path in cultured embryo-derived tissues of wheat. Previously, the introduction of a *TaAOS* gene driven by the *Ubi1* promoter to rice was also reported [45], but no information was provided concerning the transformation efficiency. The researchers produced at least six independent events of rice using the amyliglicoside-based (hygromycin) selection approach [46]. The function underlying the observed hindrance in our experiments might have relevance to the known function of AOS as the key jasmonate pathway enzyme. It has been reported that exogenously applied JA can negatively and positively affect an in vitro plant tissue culture depending on the concentration [47,48]. The affected processes include somatic embryogenesis, which is the main developmental process in cultured tissues of wheat. In Arabidopsis, the stimulation inhibition of somatic embryogenesis was found to depend on the concentration of JA. The presence of low JA concentrations in the medium can induce somatic embryogenesis, while the application of very high concentrations causes inhibition [49,50]. For this reason, the published data indicate that the supply of JA can stimulate or inhibit somatic embryogenesis [51,52], and the combined effect of concentration, cultured tissue type, and the sensitivity of the plant species should be considered [49,50].

It can be supposed that the overexpression of the *AOS* gene in wheat tissues significantly increased the endogenous concentration of jasmonates, which lead to an inhibition of somatic embryogenesis in both emmer wheat and bread wheat. An increase in jasmonate levels resulting from *AOS* overexpression may induce stress responses in tissues, and the callus cells cannot tolerate additional stress from the selective pressure of herbicide. This can explain why decreasing the concentration of the selective substances allowed the production of at least several independent transgenic plants overexpressing *TaAOS* after the reduction of additional stress factors.

Analyses of a large number of lines overexpressing one or both of the two genes *AOS* and *OPR* allowed us to identify various patterns of accumulation of certain jasmonates in hexaploid and tetraploid wheat. Among this large number of transgenic lines of hexaploid wheat, described previously [12,29] and in this article, a statistically significant increase in the level of jasmonates, specifically 12-OPDA, was observed only in the *TaAOS* overexpressors, SD2 and SD3. This may be attributed to the incorporation of the Kozak sequence into the vector upstream of the protein-coding sequence, or alternatively, to the distinctive characteristics of the wheat gene sequence, which may be inherently optimized for translation, protein transport into chloroplasts, or enzymatic activity. A comparison of the results of *AOS* gene overexpression in tetraploid and hexaploid wheat is of particular interest. We have previously shown that in transgenic tetraploid emmer plants overexpressing *AOS* from Arabidopsis (*AtAOS*), the level of 12-OPDA is not increased, while JA and JA-Ile are significantly increased [28]. Similar to these data, overexpression of the *AOS* gene from wheat (*TaAOS*) also leads in transgenic Runo plants to a significant increase in stress-induced levels of JA and JA-Ile, without changing the level of 12-OPDA. The data may suggest a higher activity of 12-OPDA transport outside of chloroplasts and/or the activity of downstream reactions of jasmonate biosynthesis occurring outside of chloroplasts in Runo. In the bread wheat Sar-60, however, these steps are seen to be the limiting factors in the biosynthesis of this hormone.

It is also noteworthy to mention a reduced basal level of JA and/or JA-Ile in plants that have been genetically modified to overexpress *TaAOS*. The same effect was previously observed in a number of transgenic lines overexpressing the *AtOPR3* gene [28]. This may be evidence of the activity of regulatory feedback loops that control the basal level of jasmonates in wheat tissues.

The increased jasmonate levels were accompanied by characteristic biological effects, including a reduction in plant height, leaf blade length, and the number of grains in the ear. Once more, as in our preceding studies, biological effects were observed not only in plants exhibiting statistically significant alterations in jasmonate levels in their leaves but also in transgenic plants that did not demonstrate notable changes in the content of these hormones relative to the non-transgenic control. This phenomenon may be explained by the fact that minor, site-specific, or transient alterations in hormone concentrations are sufficient to fulfill regulatory functions. In order to address the challenges of altering plant phenotype characteristics, such as the activation of defense pathways by phytohormones, it is essential to consider the pleiotropic nature of the induced changes. In our experiments, the activation of the jasmonate system resulted in a notable decline in the growth and production characteristics of wheat, which in some instances led to the loss of plant fertility, as observed in the RAB2 and RAB5 lines.

Jasmonates are known for their ability to regulate the defense responses of plants under unfavorable environmental conditions. However, this activation of defense mechanisms may result in the depletion of resources that are essential for plant growth and productivity, leading to significant reductions in these parameters. In order to establish a foundation for the utilization of jasmonate pathway activation in agricultural practices, it is essential to conduct a comprehensive evaluation of the impact of such activation on growth and productivity characteristics. The present study has addressed this important question.

## 4. Materials and Methods

### 4.1. TaAOS Gene Cloning and Construction of Expression Vectors

The protein-coding sequence of the *TaAOS* gene was obtained using PCR to take the genomic DNA of the wheat variety Sar-60, since this gene does not contain introns. A pair of primers was selected for the specific amplification of a “short” variant of the *AOS* gene from the wheat genome “A” (TraesCS4A02G061800.1), also known as *TaAOS2*. The sequences of the primers for *TaAOS* cloning are provided in the Table S1. To clone *TaAOS* into the vector, NotI (5′-primer) and EcoRV (3′-primer) restriction sites were added to the primer sequences. Furthermore, to facilitate the protein’s expression in plants, a Kozak sequence for monocotyledons (AACC) [53] was incorporated into the 5′-primer upstream of the initiation codon AUG. Following amplification, the *TaAOS* gene (~1450 bp) was cloned into the pENTR1A dual vector (Gateway Entry vector) at a position between the two attL elements. The absence of mutations in the cloned *TaAOS* gene was confirmed by sequencing.

**Figure 9:**
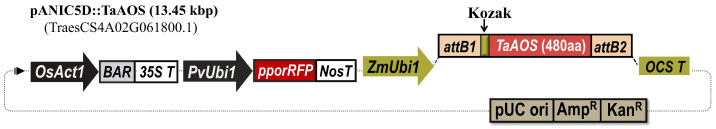
Schematic representation of the pANIC-*TaAOS* expression cassette used for emmer wheat and bread wheat transformation. *OsAct1*, rice *Actin 1* promoter; *BAR*, BASTA resistance gene (phosphinothricin acetyl transferase); 35ST, CaMV 35S terminator; PvUbi1, *Ubiquitin 1* promoter from *Panicum virgatum*; pporRFP, Red Fluorescent Protein gene from *Porites porites*; *NosT*, *Nopaline Synthase* terminator; *ZmUbi1*, maize *Ubiquitin 1* promoter; OCS T, octopine synthase terminator sequence; attB1 and attB2—site-specific recombination sequences; *Amp^R^*, ampicillin resistance gene; *Kan^R^*, kanamycin resistance gene. Arrows indicate promoters; regions controlling the expression of *TaAOS* gene are highlighted in green color.

Then, the vector for the expression of the wheat *TaAOS* gene in hexaploid and tetraploid wheat under the control of a strong constitutive promoter was generated. The transfer of the *TaAOS* gene from a donor plasmid (pENTR1A dual) into the cereal expression vector pANIC5D (ampR, kanR, bar, pporRFP) [54] was conducted using the LR clonase enzyme for Gateway cloning (Figure 9). The pANIC5D vector provides *TaAOS* gene expression under the control of a strong constitutive ubiquitin promoter from maize (*ZmUbi1*) and an octopine synthase transcription terminator (*ocsT*).

### 4.2. Generation of Transgenic Plants of Emmer Wheat and Bread Wheat

Primary transgenic plants of emmer wheat (*T. dicoccum*) of the cv. Runo and bread wheat (*T. aestivum*) of the cv. Saratovskaya-60 (Sar-60) were generated using a particle inflow gun. The pBAR-GFP.UbiAOS, pBAR-GFP.UbiOPR3, and pANIC-*TaAOS* plasmids were delivered into immature embryo-derived embryogenic calli, and putative transgenic plants were recovered using the protocol described earlier [35]. To produce ‘double’ transgenic wheat plants, the *AtAOS* gene cassette (pBAR-GFP.UbiAOS) and the *AtOPR3* gene cassette (pBAR-GFP.UbiOPR3) were co-precipitated on tungsten particles using a 1:1 molar ratio. For the screening of transgenic plantlets, an herbicide-resistance selection method was combined with the detection of fluorescent reporter gene expression using a ZEISS SteREO Discovery.V12 microscope (Carl Zeiss Microscopy GmbH, Jena, Germany) equipped with a PentaFluar S 120 vertical illuminator (Leistungselektronik JENA GmbH, Jena, Germany). All of the regenerated putative transgenic plantlets were potted into soil and grown in a greenhouse; the young fully opened leaves were used for extraction of total plant DNA and RNA as described previously [28]. T0 transformants were identified by PCR amplification of their genomic DNA using specific primers for the *GFP* or *RFP* genes, as well as primers for the *AtAOS*, *AtOPR3*, or *AtAOS* genes (Table S1), depending on the transferred vector. The primary T0 transgenic plants were further analyzed for the expression of the *AtAOS*, *AtOPR3,* or *AtAOS* genes by end-point RT-PCR using the primers listed in Table S1. The T1 to T3 generations of the transgenic plants were tracked using PCR-based genotyping; the expression of the *GFP/RFP* gene in pollen was used to identify homozygous progenies as described earlier [29]. The transcript levels of *AtAOS*, *AtOPR3,* or *AtAOS* in the homozygous T4 generation of transgenic lines were measured as described previously [27] using quantitative real-time RT-PCR with the primers listed in Table S1. Overexpression of the introduced genes was quantified with a QuantStudio™ 5 Real-Time PCR Cycler (Thermo Fisher Scientific, Waltham, MA, USA) and normalized using the *TaWIN1* housekeeping gene [55].

### 4.3. Analysis of Phytohormones

The analysis of phytohormones in transgenic and non-transgenic wheat was carried out exactly as previously described [12]. All experiments were accomplished in six independent replicas. For wounding experiments, leaf tissues were collected 30 min after wounding. Hormone extraction was performed exactly as described [12,28]. For the quantitative analysis of jasmonates, reversed phase-ultra-high performance liquid chromatography coupled on line with a triple quadrupole tandem mass spectrometry (RP-UHPLC-QqQ-MS/MS) in the multiple reaction monitoring (MRM) mode was used [56] in combination with an ACQUITY H-Class UPLC ultrahigh performance liquid chromatography system (Waters GmbH, Eschborn, Germany) coupled on line to a QTRAP 6500 (AB Sciex, Darmstadt, Germany) triple quadrupole-linear ion trap instrument operating in negative MRM mode under the instrument settings described by Leonova and co-workers [57].

### 4.4. Measurements of the Leaf Length

To analyze the effects of the introduced genes on the growth parameters, the wheat plants were grown in one-liter pots, with two plants per pot. Pots were cultivated in a glass greenhouse under controlled conditions (40–60% relative humidity; 25 ± 2 °C:20 ± 2 °C day:night temperatures; 16 h:8 h light:dark regime; light intensity up to 200 µmol m^−2^s^−1^). The measurement of the length of the 1st, 2nd, 3rd, and 4th leaves of the wheat plants was carried out as described previously [28]. The height of the plants was measured from the base to the top of the spike and represented as an average height of the three main stems. The results were analyzed by ANOVA and Dunnett’s multiple comparisons test.

## Figures and Tables

**Table 1:** Jasmonate content in intact and wounded leaves of studied wheat genotypes.

Line	12-OPDA		JA		JA-Ile	
	Intact	Wounded	Intact	Wounded	Intact	Wounded
Terraploid emmer wheat						
Runo	13.2 ± 2.2	22.2 ± 3.1	14.7 ± 2.0	38.5 ± 5.5	6.9 ± 0.7	46.4 ± 2.9
RD1	14.4 ± 2.6	24.1 ± 4.8	5.6 ± 2.1 *	78.5 ± 14.6 *	3.8 ± 0.7 *	91.3 ± 12.6 *
RD4	14.3 ± 2.9	23.7 ± 4.2	14.11 ± 4.0	67.3 ± 17.6	6.2 ± 0.6	75.3 ± 4.7 *
Hexaploid bread wheat						
Sar-60	12.3 ± 2.5	42.5 ± 20.1	7.1 ± 0.7	56.9 ± 23.4	5.1 ± 0.8	68.5 ± 25.8
SD2	48.2 ± 20.6	159.6 ± 51.8 *	4.8 ± 1.6	72.7 ± 10.9	5.0 ± 0.7	94.3 ± 7.1
SD3	29.3 ± 5.7 *	238.7 ± 56.9 *	3.3 ± 1.0 *	65.6 ± 9.6	5.6 ± 1.0	153.3 ± 44.0

Data are averages of six measurements ± standard error. Statistically significant difference (*, *p* < 0.05) in metabolite level from non-transgenic plants is marked by color, red—decreased level, and green—increased level.

**Table 2:** The efficiency of genetic transformation of emmer wheat (cv. Runo) and bread wheat (cv. Sar-60) with the JA biosynthesis genes encoding ALLENE OXIDE SYNTHASE (*AtAOS* or *TaAOS*) and OXOPHYTODIENOATE REDUCTASE (*AtOPR3*).

Cultivar	Gene(s)	Number of Explants		T0 Events		Transformation Efficiency (%)		Reference
		Bombarded	Produced Transgenic T0 Plants (GFP/RFP+)	Carrying Insertion of JA Biosynthesis Gene (PCR+)	Overexpression of JA Biosynthesis Gene (RT-PCR+)	Selective Genes	JA Biosynthesis Genes	
Runo (emmer wheat)	*AtAOS* *	576	10	10	6	1.7	1.7	[12]
	*AtOPR3* *	236	31	30	26	13.1	12.7	[12]
	*AtAOS* + *AtOPR3* *	301	10	6	3	3.3	2.0	this study
	*TaAOS* **	547	3	3	3	0.5	0.5	this study
	Empty vector *	653	85	-	-	13.0	-	[35]
Sar-60 (bread wheat)	*AtAOS* *	717	8	7	4	1.1	1.0	this study
	*AtOPR3* *	377	16	15	9	4.2	4.0	[29]
	*AtAOS* + *AtOPR3* *	358	3	3	3	0.8	0.8	this study
	*TaAOS* **	1013	3	3	3	0.3	0.3	this study
	Empty vector *	420	11	-	-	2.6	-	[29]

* The expression cassette includes *BAR* and *GFP* selective genes, ** The expression cassette includes *BAR* and *RFP* selective genes.

## Data Availability

The presented data are available upon reasonable request from the corresponding author.

## Supplementary Materials

The following supporting information can be downloaded at: https://www.mdpi.com/article/10.3390/plants13192781/s1, Table S1: List of primers used in the study; Figure S1: Analysis of putative transgenic plants of emmer wheat Runo (*T. dicoccum*) for integration and expression of transgenes from pBAR-GFP.UbiAOS and pUbiOPR3 vectors; Figure S2: Analysis of putative transgenic plants of bread wheat Sar-60 (*T. aestivum*) for integration and expression of transgenes from pBAR-GFP.UbiAOS and pUbiOPR3 vectors; Figure S3: Analysis of putative transgenic plants of bread wheat Sar-60 (*T. aestivum*) for integration and expression of transgenes from pBAR-GFP.UbiAOS vector; Figure S4: Analysis of putative transgenic plants of bread wheat Sar-60 (*T. aestivum*) and emmer wheat Runo (*T. dicoccum*) for integration of transgenes from pANIC-*TaAOS* vector; Figure S5: Jasmonate content in intact (light gray) and wounded (dark gray) leaves of transgenic Sar-60 (*T. aestivum*) plants overexpressing *AtAOS* (SA7) or two genes *AtAOS* and *AtOPR3* (SAB1 and SAB3) from *A. thaliana*; Figure S6: Jasmonic acid content in intact leaves of transgenic Runo (*T. dicoccum*) plants overexpressing two Arabidopsis genes *AtAOS* and *AtOPR3*.
