# Supplementary material for: Genetic Transformation of Triticum dicoccum and Triticum aestivum with Genes of Jasmonate Biosynthesis Pathway Affects Growth and Productivity Characteristics
Source: Plants (Basel). 2024 Oct 4;13(19):2781. doi: 10.3390/plants13192781 (PMC11478715; doi:10.3390/plants13192781)
Supplement: Supplementary file 1 [file plants-13-02781-s001.zip › plants-3221730-supplementary.pdf]

Supplementary Material

Genetic transformation of *Triticum dicoccum* and *Triticum aestivum* with genes of jasmonate biosynthesis pathway affects growth and productivity characteristics

Dmitry N. Miroshnichenko*, Alexey V. Pigolev, Alexander S. Pushin, Valeria V. Alekseeva, Vlada I. Degtyaryova, Evgeny A. Degtyaryov, Irina V. Pronina, Andrej Frolov, Sergey V. Dolgov, and Tatyana V. Savchenko

Supplementary Table 1. List of primers used in the study

Primer name	Sequence (5′ – 3′)	Gene	Product length, bp	Purpose
TaWIN-for	TTTTCTGTGTTCTACTATGAGATCTTGAA	*TaWIN1*	348	RT-PCR, qPCR
TaWIN-rev	AAGTGCATAATTAAACAGAGGTAGTGATG			
AtAOSbF	AAATCCAACGGCGGAGAACT	*AtAOS*	100	qPCR
AtAOSbR	TCGTCGCCAACGGTTGATAA			
AtOPR3mid-F	CCGAAGGCACCATGGTCTCTCCC	*AtOPR3*	295	PCR, RT-PCR
AtOPR3mid-R	TCGGAAGCTTCTAAAGCCCGAGG			
AtOPR3a-F	ACGTGCTTCTCATGCAGTGT	*AtOPR3*	100	qPCR
AtOPR3a-R	ACTTCACGTGGGAACCATCG			
GFP-For	GCGACGTAAACGGCCACAAG	*sGFP*	606	PCR, RT-PCR
GFP-Rev	CCAGCAGGACCATGTGTGATCG			
TaAOSshbF<br>TaAOSshbR	GGCCGGAGAGAAGTTCCAC<br>CTTCTCCAGCGCCTCTATCG	*TaAOS*	100	qPCR
AtAOSc dir	GAATCCGCCGGTGAGATTCTCGTTGAAGCCG	*AtAOS*	664	PCR
AtAOSc rev	ACGAGAAATTAACGGAGCTTCCTAACGGCGACG			
PropRFP for	GTCAAAGATGTAATGAACACCGAGCTTC	*RPF*	658	PCR RT-PCR
PropRFP rev	AGTTAGAAAGATGTGCATGTGCAATTTCTC			
TaAOS sh3 for	CAAGGCCGACATGAACATCGAGA	*TaAOS*	492	PCR
3'UTR AOS sh rev	CTAGTCCCGGGTCTTAATTAACTCTCTAGACTC			

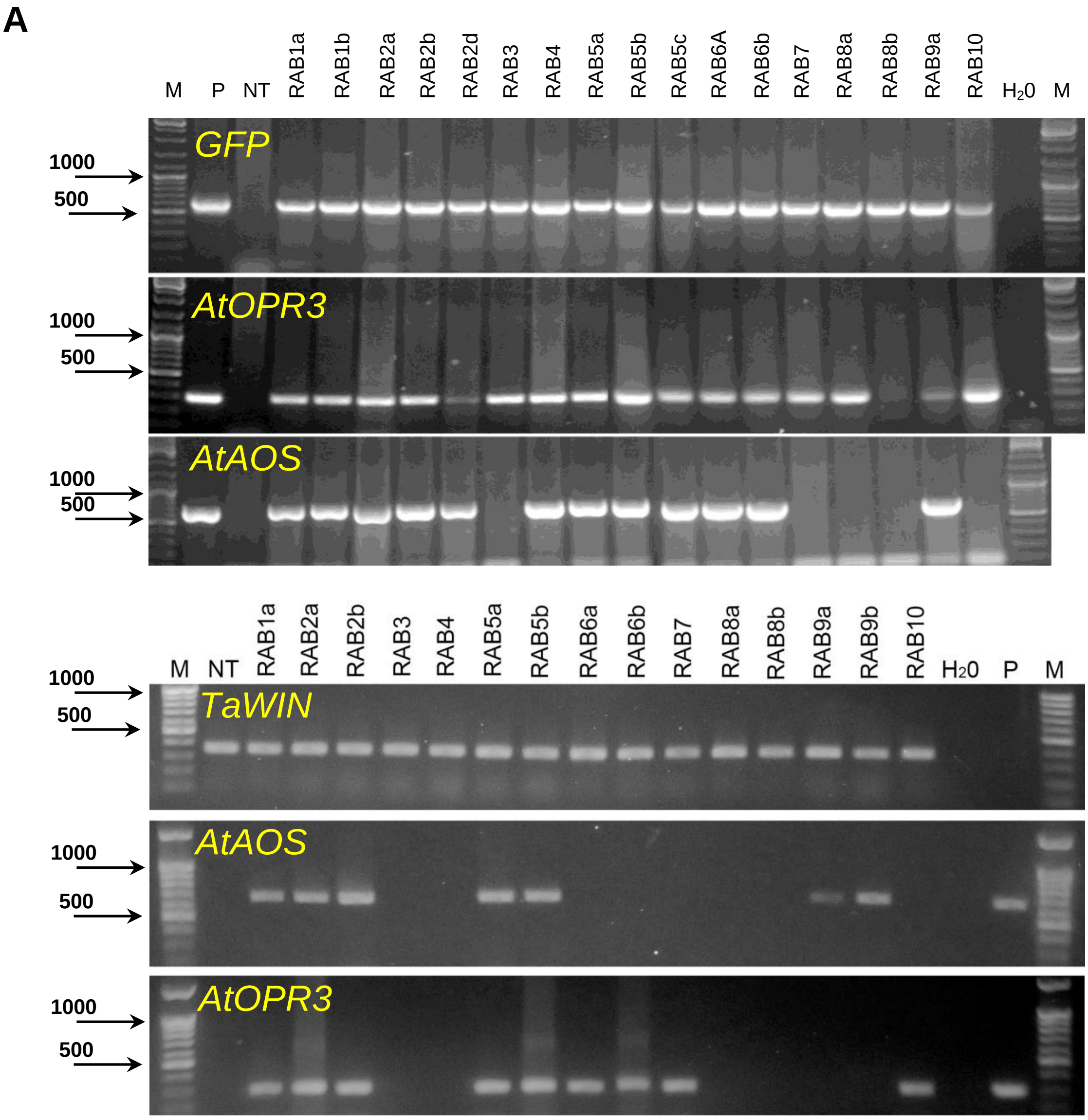

Figure S1. Analysis of putative transgenic plants of emmer wheat Runo (*T.dicoccum*) for integration and expression of transgenes from pBAR-GFP.UbiAOS and pUbiOPR3 vectors. (**A**), PCR analysis for integration of sequences encoding *GFP* (top panel), *AtOPR3* (middle panel) and *AtAOS* (bottom panel) genes into genome of T0 plants. (**B**), End-point RT-PCR analysis on the total RNA of T0 plants (at heading stage) for the expression of the *TaWIN1* (top panel), *AtOPR3* (middle panel) and *AtAOS* (bottom panel) genes. Lane M, DNA ladder as a molecular weight marker; Lane P, DNA of corresponding vector; Lane NT, non-transgenic wheat plant Runo, Lanes labelled 1–10 represent putative transgenic wheat plants established in greenhouse; in some cases, two plantlets (indicated as *a*, or *b*, or *c*, or *d*) were regenerated from one explant, and plantlets were independently analysed for insertion and expression of transgenes.

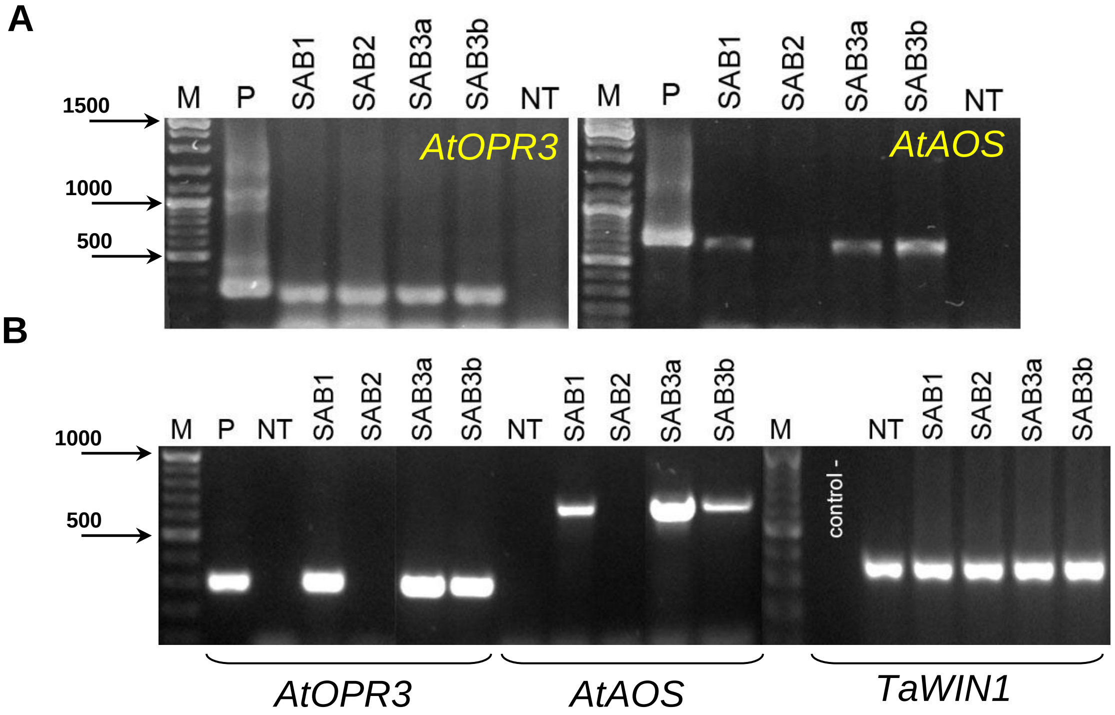

Figure S2. Analysis of putative transgenic plants of bread wheat Sar-60 (*T. aestivum*) for integration and expression of transgenes from pBAR-GFP.UbiAOS and pUbiOPR3 vectors. (**A**), PCR analysis for integration of sequences encoding *AtOPR3* (right panel) and *AtAOS* (left panel) genes into genome of T0 plants. (**B**), End-point RT-PCR analysis on the total RNA of T0 plants (at heading stage) for the expression of the *AtOPR3, AtAOS* and *TaWIN1* genes. Lane M, DNA ladder as a molecular weight marker; Lane P1, plasmid DNA pBAR-GFP.UbiOPR3; Lane P2, plasmid DNA pBAR-GFP.UbiAOS; Lane NT, non-transgenic wheat plant Sar-60, Lanes labelled 1–3 represent putative transgenic wheat plants established in greenhouse; in some cases, two plantlets (indicated as *a,* or *b*) were regenerated from one explant, and plantlets were independently analysed for insertion and expression of transgenes.

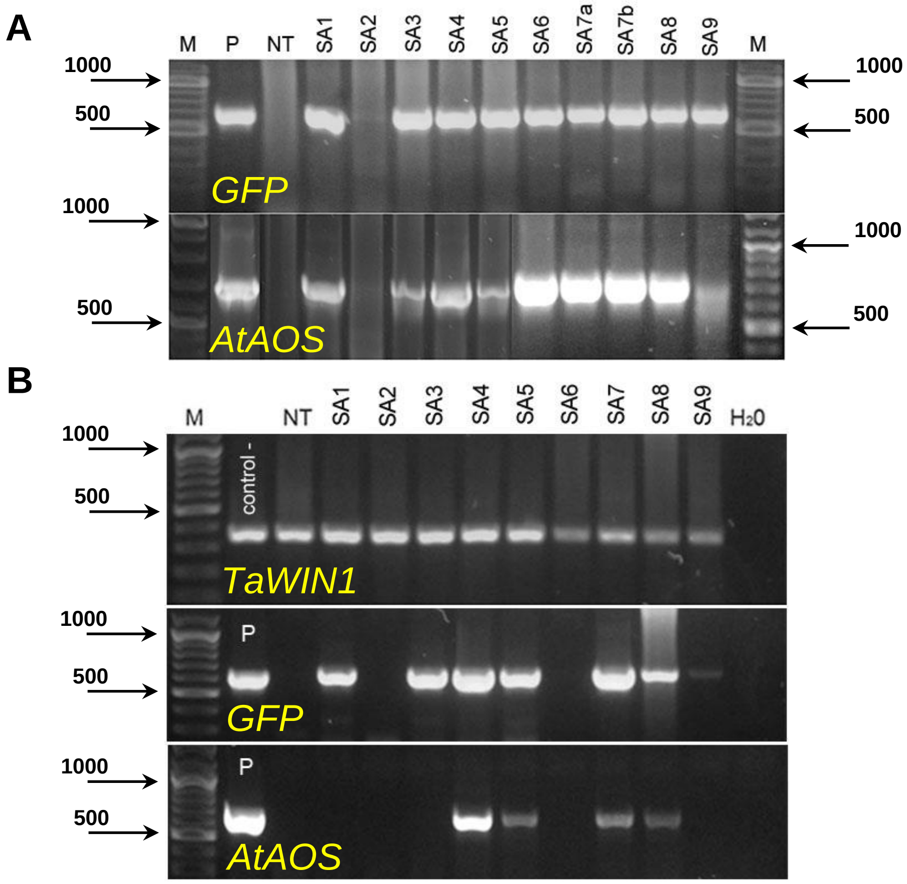

Figure S3. Analysis of putative transgenic plants of bread wheat Sar-60 (*T. aestivum*) for integration and expression of transgenes from pBAR-GFP.UbiAOS vector.

(**A**), PCR analysis for integration of sequences encoding *GFP* (top panel) and *AtAOS* (bottom panel) genes into genome of putative transgenic plants. (**B**), End-point RT-PCR analysis on the total RNA of T0 plants (at heading stage) for the expression of the reference gene *TaWIN1* (top panel), *GFP* gene (middle panel) and *AtAOS* (bottom panel). Lane M, DNA ladder as a molecular weight marker; Lane P, plasmid DNA of pBAR-GFP.UbiAOS; Lane NT, non-transgenic wheat plant Sar-60, Lanes labelled 1–9 represent putative transgenic wheat plants established in greenhouse; in some cases, two plantlets were regenerated from one explant, and plantlets were independently analysed for transgenes insertion and expression (indicated as *a* or *b*).

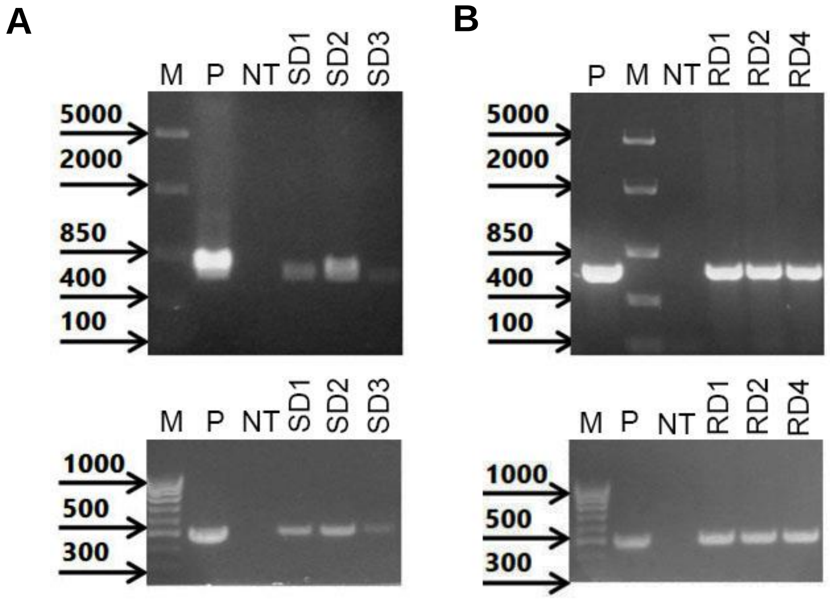

Figure S4. Analysis of putative transgenic plants of bread wheat Sar-60 (*T. aestivum*) (**A**) and emmer wheat Runo (*T. dicoccum*) (**B**) for integration of transgenes from pAnic-TaAOS vector. (**A**), PCR analysis for integration of sequences encoding *RFP* (top panel) and *TaAOS* (bottom panel) genes into genome of T0 plants of 'Saratovskaya-60'. (**B**), PCR analysis for integration of sequences encoding *RFP* (top panel) and *TaAOS* (bottom panel) genes into genome of T0 plants of 'Runo'. Lane M, DNA ladder as a molecular weight marker; Lane P, plasmid DNA of pANIC-TaAOS; Lane NT, non-transgenic wheat plant, Lanes labelled 1–4 represent putative transgenic wheat plants established in greenhouse; in some cases, two plantlets (indicated as *a*, or *b*) were regenerated from one explant, and plantlets were independently analysed for insertion of transgenes.

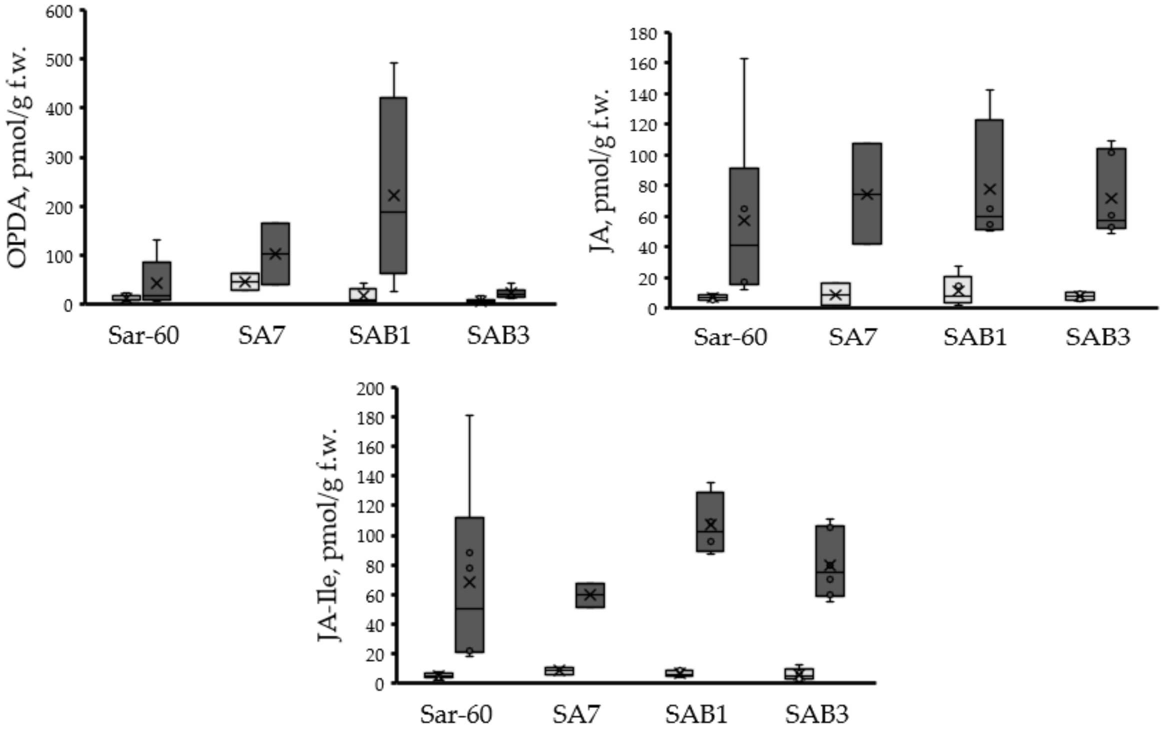

Figure S5. Jasmonate content in intact (light gray) and wounded (dark gray) leaves of transgenic Sar-60 (*T. aestivum*) plants overexpressing *AtAOS* (SA7) or two genes *AtAOS* and *AtOPR3* (SAB1 and SAB3) from *A. thaliana*. Each box represents data from six biological replicates with whiskers extended to the extreme data points; the midline is the median, the cross is the mean.

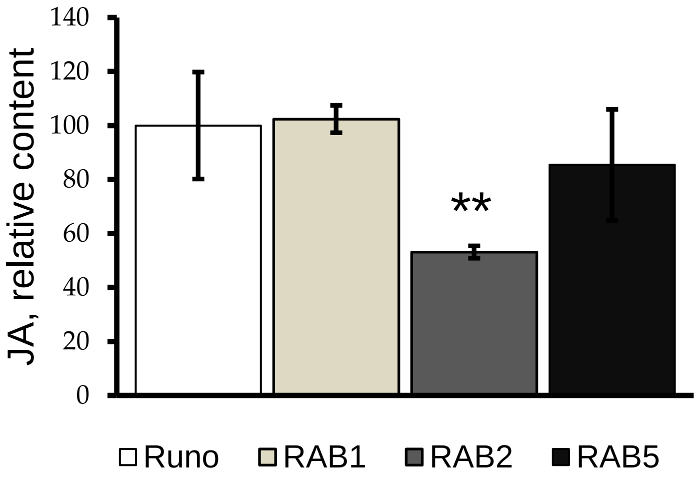

Figure S6. Jasmonic acid content in intact leaves of transgenic Runo (*T.dicoccum*) plants overexpressing two Arabidopsis genes *AtAOS* and *AtOPR3*. "**" indicates statistically significant difference between transgenic line and non-transgenic control at p≤0.01, determined by Student's t-test.
